# Efficacy and safety of Bruton's tyrosine kinase inhibitors in the treatment of pemphigus: A comprehensive literature review and future perspective

**DOI:** 10.1016/j.heliyon.2023.e22912

**Published:** 2023-11-27

**Authors:** Yekta Ghane, Nazila Heidari, Amirhossein Heidari, Sara Sadeghi, Azadeh Goodarzi

**Affiliations:** a School of Medicine, Tehran University of Medical Sciences, Tehran, Iran; b School of Medicine, Iran University of Medical Sciences, Tehran, Iran; c Faculty of Medicine, Tehran Medical Sciences, Islamic Azad University, Tehran, Iran; d Department of Medicine, New York Health System, South Brooklyn Hospital, NY, USA; e Department of Dermatology, Rasool Akram Medical Complex Clinical Research Development Center (RCRDC), School of Medicine, Iran University of Medical Sciences, Tehran, Iran

**Keywords:** Bruton's tyrosine kinase inhibitors, Immunomodulator agents, Pemphigus, Treatment, Bruton's tyrosine kinase

## Abstract

Bruton's tyrosine kinase (BTK) is a protein involved in B-cell-receptor signaling and B-cell proliferation. The pathophysiology of several autoimmune diseases, such as pemphigus disorder, relies on the BTK signaling pathway. Therefore, BTK inhibitors were found to be beneficial alternatives to conventional treatmentsThe current study aimed to assess the efficacy and safety of BTK inhibitors in treating pemphigus. A complete search was performed on databases including PubMed/MedLine, Scopus, Web of Science, as well as Google Scholar search engine for studies published by September 20th, 2023. The current review indicates that BTK inhibitors alone or in combination with conventional treatments are promising options in the management of pemphigus. The overall safety profile of BTK inhibitors has been acceptable, and the reported adverse reactions were not severe or life-threatening.

## Highlights

• Pemphigus is a group of immunobullous disorders involving the mucocutaneous membranes' desmosomes with high mortality and morbidity rates.
• Previous treatment options, such as high-dose oral corticosteroids and rituximab, were found to be associated with considerable adverse events with uncertain safety and efficacy.
• Bruton Tyrosine Kinase (BTK) plays an important role in several immunological pathways; hence, they are potential treatments for certain autoimmune disorders.
• Administration of reversible BTK inhibitors alone or in addition to conventional treatments can be promising combinations in managing pemphigus due to their high response rate, mild and limited side effects, and acceptable results in terms of safety and efficacy.

## 1 Introduction

Pemphigus is a chronic, devastating autoimmune blistering disorder affecting the skin and mucous membranes [1]. The major types of pemphigus are pemphigus vulgaris (PV), pemphigus foliaceous (PF), and rarely paraneoplastic pemphigus (PNP), Immunoglobloin (Ig) A pemphigus, and drug-induced pemphigus [2]. Despite the low prevalence of the disease worldwide, pemphigus is a life-threatening condition with high morbidity and mortality rates [3]. The mechanism of pemphigus disorder is based on the activity of IgG autoantibodies that target the adhesion proteins of the epidermis: desmoglein (Dsg)-1 and Dsg-3 [1,4]. The process results in loss of epidermal keratinocyte adhesion (acantholysis), intraepidermal blistering, and erosions with poor prognosis.

Several treatments have been administrated to manage pemphigus, including high-dose oral corticosteroids (CSs) (first line), plasma exchange, and nontargeted immunomodulating agents such as intravenous immunoglobulin (IVIG), azathioprine, mycophenolate, and cyclophosphamide [3,4]. However, the efficacy and safety of such treatments are not completely certain. Over the last decade, rituximab has been suggested as a new therapeutic option for pemphigus diseases [4,5]. By detail, rituximab is an anti-CD20 monoclonal antibody and is utilized to manage patients who did not respond to conventional treatments. Despite this, anti-drug antibodies such as rituximab may cause a paradoxical exacerbation, loss of efficacy, or infusion reactions during the pemphigus treatment [5].

Bruton's tyrosine kinase (BTK)-dependent signaling pathway is claimed to have a significant role in the pathogenesis of autoantibody-driven dermatological conditions like pemphigus [6]. BTK is an enzyme involved in the B-cell-receptor signaling pathway and B-cell proliferation, with an effect on both innate and adaptive immune responses [4,7]. Based on evidence, the use of BTK inhibitors in treating immune-mediated conditions has been increasingly investigated [8]. Ibrutinib is an irreversible BTK inhibitor that was first suggested to be beneficial to treat PNP in patients with underlying B cell malignancy [9,10]. Rilzabrutinib and tirabrutinib are reversible and selective oral BTK inhibitors that are considered for treating pemphigus and have shown acceptable safety and efficacy profiles [4,8].

In this study, we aim to provide a comprehensive review of current evidence and future prospects of BTK inhibitors in the treatment of different types of pemphigus. We declare the mechanism of action, safety, efficacy, challenges, and side effects of BTK inhibitors as novel therapeutic options in patients with pemphigus.

## 2 Materials and method

A thorough search was performed on PubMed (Medline), Scopus, and Web of Science databases, as well as the Google Scholar search engine, using related MeSH words and key terms to find relevant studies by September 20th, 2023. Regarding screening the articles, original publications with related topics, available full text, and English language were included.

## 3 Results

### 3.1 Characteristics of eligible studies

**Fig. 1:**
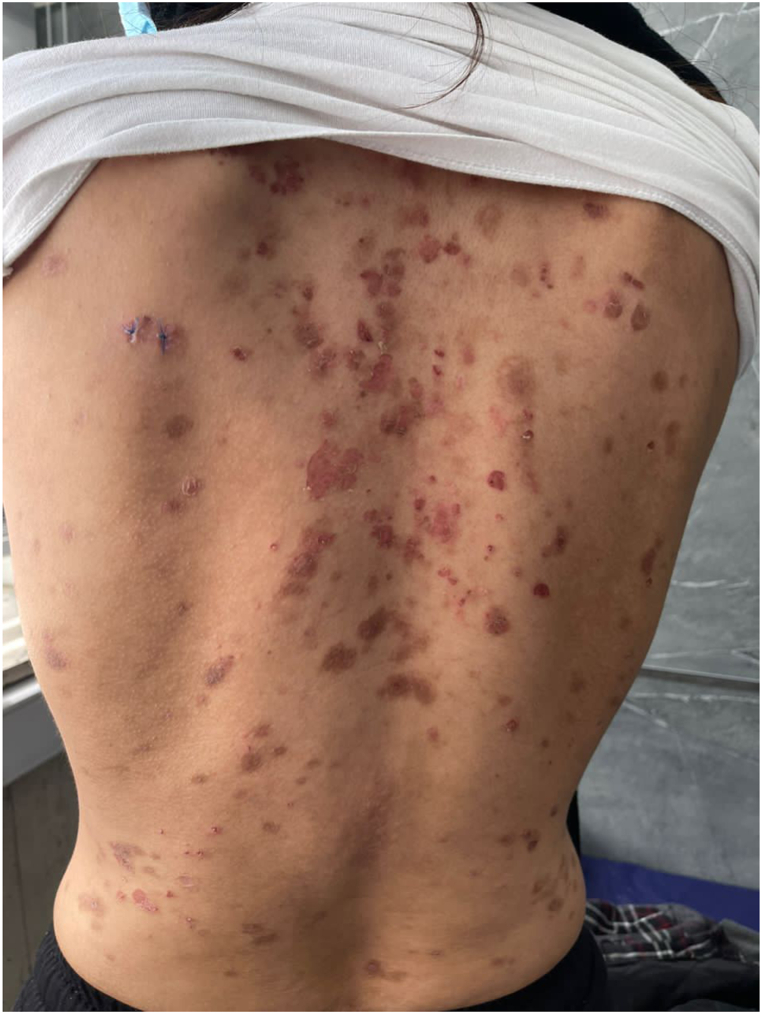
Leafy and crusted circumscribed erosion on the back of a case of PF retrieved by AG (corresponding author) from her clinic.

**Fig. 2:**
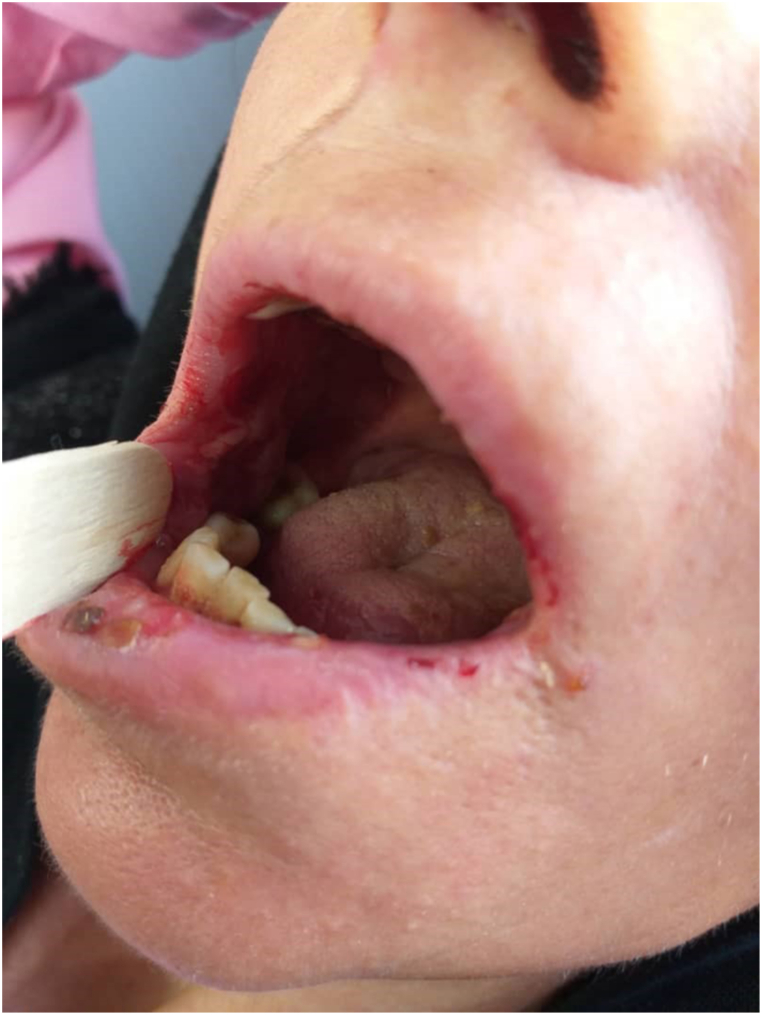
Oral lesions in a case of PV retrieved by AG (corresponding author) from her clinic.

A total of six studies consisting of two clinical trials, two case reports, and two experimental studies have been selected to be included in this article. Characteristics of eligible studies were completely illustrated in Table 1. Moreover, two cases of PF and PV are demonstrated in Fig. 1, Fig. 2, respectively.

**Table 1:** Evaluation of clinical and pre-clinical studies on BTK inhibitors in pemphigus diseases.

Study ID	Study Design	Sample Size	Age (Mean)	Gender Ratio (M: male)	Type of Disease	Previous Treatment	BTK Inhibitor Treatment	Treatment Duration (weeks)	Outcome Measurement	Efficacy	Adverse events	Comorbidities	Follow-up Evaluation
Goodale, 2020 [11]	Experimental	9	8.2	M: 56%	Canine pemphigus foliaceus	None	Atuzabrutinib 15 mg/kg daily in combination with PRN prednisolone 1 mg/kg twice a day	16–20	Physical examination, anti-Dsc-1 titer, and Wilcoxon rank sum test	33% near-complete remission 44% poor or fair response despite initial improvement	Recurrence of a previously excised mast cell tumor (11%), lymphadenopathy, and Immune-mediated polyarthritis (22%)	33% severe allergic dermatitis, 11% mast cell tumor, 11% chronic pancreatitis	Relapse following treatment break from BTKi
Goodale, 2020 [12]	Experimental	4	9.25	M: 25%	Canine pemphigus foliaceus	None	Rilzabrutinib, 17–33 mg/kg	20	cPDAI, Serum anti-Dsc-1, and Dsg-1 IgG titers	75% near-complete remission 25% fair response	Pyometra (25%)	Not mentioned	Not mentioned
Lee, 2017 [9]	Case report	1	51	M	Paraneoplastic pemphigus	Oral Corticosteroid, intravenous Corticosteroid, and Rituximab	Ibrutinib 420 mg daily in combination with rituximab	16	Physical examination and clinical evaluation	Decrease signs and symptoms, especially cutaneous manifestations	Florid ecchymosis and purpura	CLL	Clinical remission after total taper after 3 months
Murrell, 2021 [13]	Clinical trial Phase II	27	51	M: 44%	Moderate to severe pemphigus Vulgaris	High-dose corticosteroids and rituximab	Rilzabrutinib 400–600 mg twice daily with or without low-dose corticosteroid	12	Primary endpoint: CDA within 4 weeks. Secondary endpoint: time to receive CDA, complete remission, relapse post rilzabrutinib, and corticosteroid usage. PDAI score, anti-dsg3 antibody level	Primary endpoint CDA; 52%, decrease mean corticosteroid dose, 22% complete remission by week 24, Improve PDAI score Decrease anti-Dsg3 antibody level	Nausea (15%), upper abdominal pain (11%), headache (15%), cellulitis grade 3 (n = 1),	Diabetes mellitus type 2 (n = 1), congenital pulmonary sequestration (n = 1)	12 weeks, successful treatment with Rilzabrutinib alone without moderate to high dose corticosteroids after 12 weeks
Ito, 2018 [10]	Case report	1	62	M	Paraneoplastic pemphigus	Bendamustine combined with rituximab	Ibrutinib 420 mg daily	Not mentioned	Physical examination and clinical evaluation	Decrease in signs and symptoms	Not mentioned	B-CLL/SLL	Not mentioned
Yamgami, 2021 [4]	Clinical trial Phase II	16	52.5 ± 8.8	M: 50%	Pemphigus vulgaris (50%), Pemphigus foliaceus (37.5%), and pemphigus vegetans (12.5%)	Oral corticosteroid and adjuvant therapy	Postprandial oral tirabrutinib 80 mg once daily	52	Primary endpoint: complete remission after 24 weeks. Secondary endpoint: absolute remission rate over time, remission rate over time, and change in PDAI score, anti-Dsg1 and Dsg3 antibody, and oral corticosteroid exposure over time	Complete remission rate:18.8% after 24 weeks and 50% after 52 weeks Decrease mean prednisolone dose over time Decrease anti-Dsg1 and Dsg3 titers baseline. Decrease PDAI score	Nasopharyngitis (n = 5), influenza (n = 3), pemphigus (n = 3), hypertension (n = 3), folliculitis (n = 2), oral candidiasis (n = 2, hepatic enzyme increase (n = 2), adverse drug reaction: nasopharyngitis (n = 3)	Not mentioned	Not mentioned

B-CLL/SLL: B-cell chronic lymphocytic leukemia/small lymphocytic lymphoma; CDA: control of disease activity; CLL: Chronic lymphocytic leukemia; Dsc: desmocollin; Dsg: desmoglein; Ig: immunoglobulin; PDAI: Pemphigus disease area index; mg: milligram; PRN: pro re nata.

### 3.2 Pre-clinical and animal studies

BTK signaling pathway has been detected in the pathogenesis of pemphigus. Some pre-clinical studies evaluated the effectiveness of BTK inhibitors in treating pemphigus in animal models. Atuzabrutinib attachment to the BTK, both covalently and noncovalently, impedes the innate and adaptive autoimmune responses [14]. Goodale et al. designed a study to demonstrate the effect of Atuzabrutinib on PF outcomes in canines [11]. Atuzabrutinib was started with 15 mg/kg once daily, then was increased to twice daily for a maximum of 20 weeks. Canine Pemphigus disease activity index (cPDAI), the titers of anti-desmocollin (Dsc)-1, anti-Dsg-1, and IgG were assessed. By detail, at the end of the fourth week, four canines demonstrated continued improvement, with three achieving a state of near-complete remission at the end of the study. Among the evaluated parameters, the anti-Dsc-1 IgG titer exhibited a decrease in three canines and an increase in two canines, remained undetectable in three canines, and was not assessed in the canine that had been withdrawn from the study. Notably, none of the canines exhibited detectable IgG antibodies against Dsg-1 in both pre and post-treatment. Five canines out of nine showed a favorable response to the medication. Subsequently, atuzabrutinib may be considered as adjuvant therapy for the treatment of PF.

Rilzabrutinib is another BTK inhibitor that decreases the migration of neutrophils and the production of autoantibodies. In another study by Goodale et al., rilzabrutinib was administered to four dogs suffering from canine PF [12]. The dosage and duration of the treatment were the same as atuzabrutinib in the previous experiment. In the initial two weeks of treatment, all four canines exhibited a decline in both lesion severity and their cPDAI scores. Among these, three dogs continued to experience improvement and achieved a sustained state of near-complete remission by the 20-week mark. The final prescribed daily dosages fell within the range of 17–33 mg/kg. In terms of anti-Dsg-1 and IgG titers, a significant reduction was observed in one dog, while undetectable levels were noted in two others, with one dog's results proving inconclusive. It is noteworthy that none of the dogs exhibited detectable IgG antibodies directed against Dsg-1. Overall, three out of four dogs showed near-complete remission, and the results claimed the effectiveness of rilzabrutinib as monotherapy.

### 3.3 Clinical studies

#### 3.3.1 Ibrutinib

Ibrutinib is an FDA-approved irreversible BTK inhibitor with considerable potential in treating B-cell malignancies [15]. Lee et al. reported a 51-year-old male patient with chronic lymphocytic leukemia (CLL) and secondary paraneoplastic pemphigus. The patient was under treatment with corticosteroids and rituximab. Ibrutinib was added to his therapy regimen, aiming to manage his progressive CLL, and interestingly, it resulted in ameliorating the PNP lesions [9]. Furthermore, Ito et al. assessed the combination of rituximab and ibrutinib in treating PNP. The result was significantly impressive in the reduction of blistering erosions in a 62-year-old male with B-cell chronic lymphocytic leukemia/small lymphocytic lymphoma (B-CLL/SLL)- associated PNP [10].

#### 3.3.2 Rilzabrutinib

As opposed to ibrutinib, rilzabrutinib is a potent BTK inhibitor with a reversible covalent binding that could boost its safety. The results of the Smith et al. phase I clinical trial depicted that single and multiple doses of oral rilzabrutinib were well-tolerated with no serious adverse events in 62 healthy participants [16]. Afterward, a phase II clinical trial was conducted by Murrell et al., assessing the effectiveness and safety of oral rilzabrutinib in individuals diagnosed with PV [13]. In this study, 27 patients diagnosed with PV were included, comprising nine newly diagnosed individuals (33%) and 18 who had experienced relapses (67%). Among these patients, 11 had moderate disease severity (41%), while 16 presented with moderate to severe disease (59%). The primary study endpoint, which was the control of disease activity (CDA) characterized by the absence of new lesions and the healing of existing ones, was attained by 14 patients, constituting 52% of the cohort (with a 95% confidence interval ranging from 32% to 71%). Notably, 11 of these patients achieved CDA through the use of low-dose CS, while three patients achieved it without the use of CS. Throughout the 12-week treatment period, the mean daily doses of CS were notably reduced. Specifically, for newly diagnosed patients, the mean CS dose decreased from 20.0 to 11.8 mg per day, while for relapsing patients, it decreased from 10.3 to 7.8 mg per day. By week 24, six patients (22%) had achieved a complete response, with four of them (15%) reaching this milestone by week 12. The majority of adverse events related to treatment were mild (grade 1 or 2), with only one patient experiencing grade 3 cellulitis. Considering these results, rilzabrutinib with concomitant low-dose corticosteroids yielded a safe and high response rate in treating patients with PV.

#### 3.3.3 Tirabrutinib

Tirabrutinib is a highly selective BTK inhibitor that has been newly utilized in treating primary lymphoma of the central nervous system, Waldenstrom macroglobulinemia, and plasma cell lymphoma [17]. Tirabrutinib creates an irreversible covalent binding with BTK that prevents inducing the IgG-autoantibody-mediated signaling pathway. In phase II of a clinical trial, 16 patients with refractory pemphigus received oral tirabrutinib 80 mg once daily for 52 weeks [4]. At the 52-week, the results demonstrated the following remission rates among the participants: complete remission was observed in 50.0% (7 out of 14), partial remission in 14.3% (2 out of 14), and an overall remission rate of 64.3% (9 out of 14). When specifically examining patients with different forms of pemphigus, namely PV, PF, and vegetans, the complete remission rates at week 52 were as follows: 57.1% (4 out of 7) for PV, 33.3% (2 out of 6) for PV, and 100.0% (1 out of 1) for vegetans. Correspondingly, the remission rates for these specific subtypes were 57.1% (4 out of 7), 66.7% (4 out of 6), and 100.0% (1 out of 1), respectively. It is noteworthy that Dsg-1 and Dsg-3 antibody titers decreased from their baseline levels while IgG levels remained stable. Additionally, a significant decrease in CS dose for controlling the disease was reported. Although the incidence of adverse effects and adverse drug reactions was 87.5 % (14 patients) and 43.8% (seven patients), respectively, there was no correlation between tirabrutinib and serious adverse effects. In detail, only two patients discontinued the treatment due to adverse events that were not attributed to tirabrutinib; one was because of gastric cancer, and the other because of pemphigus. The findings suggested that tirabrutinib can be considered a promising therapeutic choice for pemphigus.

## 4 Discussion

Pemphigus is a kind of bullous autoimmune disorder in which the autoimmune system targets its own desmosomes (mostly Dsg-1 and Dsg-3) of the mucocutaneous membranes by autoantibodies, resulting in loss of connectivity between keratinocytes. All types of pemphigus affect both humoral and cellular immunity [2,18]. The presentation of symptoms depends on the type of damaged Dsg, and symptoms may revolve without treatments in patients.

The treatment of pemphigus mainly aims to prevent the formation of new lesions, cure existing lesions, and reduce drug adverse effects. The guidelines advocate a structured approach to pemphigus treatment, comprising induction and maintenance phases [19]. The induction phase commences with the initiation of systemic CS therapy and extends until disease control is achieved, typically around two weeks after treatment initiation. The primary objective during this phase is to attain “disease control,” defined as the epithelialization of the majority of existing lesions with a concomitant absence of new blister formation through intensive and adequate therapeutic interventions [20]. In cases where the therapeutic response is deemed insufficient within the initial two-week timeframe, the necessity for supplementary treatment should be carefully considered. Given the potential risks of adverse effects, it is important to avoid the continuation of oral CS at the same dosage without a clearly defined therapeutic goal. Diabetes, hypertension, gastrointestinal bleeding and ulcerations, myopathy, osteoporosis, osteonecrosis, infection, and death have been linked to chronic administration of CS [21]. Therefore, limiting CS dosage and treatment duration is extremely critical to minimize adverse effects.

As potential supplementary treatments, the guidelines enumerate options such as immunosuppressive drugs, including azathioprine, cyclophosphamide, mycophenolate mofetil, dapsone, methotrexate, and rituximab in treating pemphigus [22]. Moreover, monoclonal antibodies against tumor factor necrosis alpha (TNF-α), such as infliximab, can be promising medications in treating pemphigus. In addition, IVIG, plasmapheresis, and immunoadsorption have been proposed as effective adjuvant therapies for severe pemphigus. Notably, some studies have suggested that Janus kinase (JAK) inhibitors may be potential modalities in the management of refractory pemphigus [5]. However, it is crucial to acknowledge that, due to their mechanism of action, immunosuppressive agents may not be optimal for swiftly controlling the rapid expansion of blisters and erosions, as they typically require over a month to reduce autoantibody levels and, subsequently, mitigate pemphigus symptoms in patients. Furthermore, if immunosuppressive agents are initiated from the outset of treatment, they may not be considered an adjunctive treatment option [19].

Protein kinase enzymes phosphorylate other proteins and exploit their substrates to activate a certain pathway. Among various types of kinases, the Tec family belongs to the non-receptor protein-tyrosine kinases family, illustrated by its first member, Tec [23]. The pleckstrin homology (PH) domain is specific to this family of receptors. Tec family includes five members: (I) tyrosine kinase expressed in hepatocellular carcinoma (TEC), (II) interleukin-2-inducible T cell kinase (ITK), (III) resting lymphocyte kinase (RLK), (IV) bone marrow expressed kinase (BMX), and (V) BTK. As a cytoplasmic enzyme, BTK is temporarily connected to the plasma membrane due to the interaction between its PH domain and phosphatidylinositol 3-phosphate (PIP3), which acts as a substantial mediator of B cell receptor (BCR) and Fc receptor signaling [15].

**Fig. 3:**
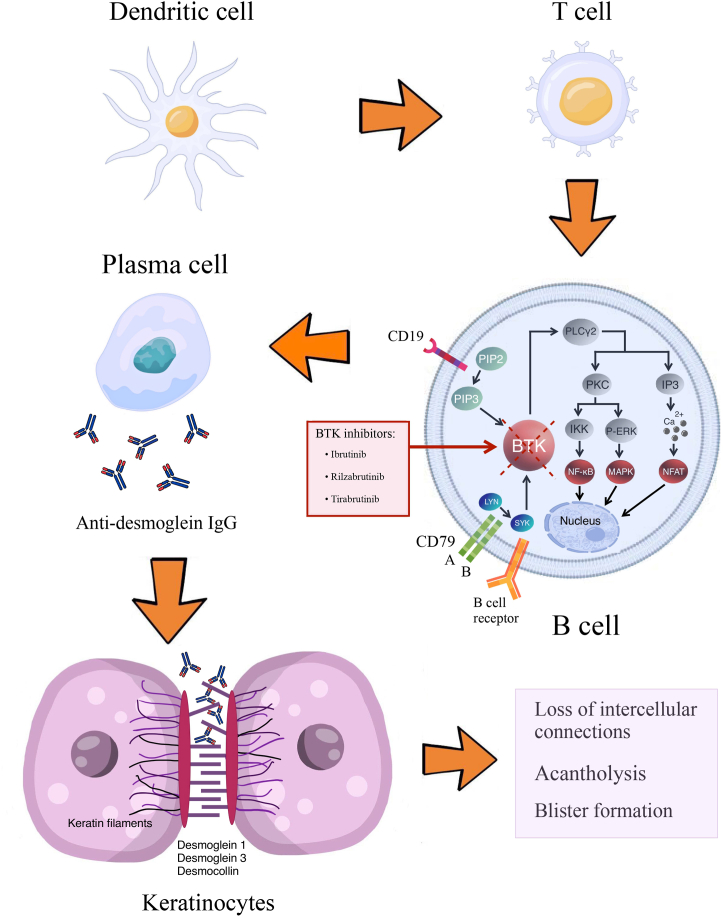
Pathophysiology of pemphigus - molecular targets for bruton tyrosine kinase inhibitors.

BTK plays an essential role in the development and survival of B cells. Subsequently, it results in activating other immune cells, increasing the production of cytokines, producing immunoglobulins by activation of phospholipase Cγ2 (PLCγ2), creating nuclear factor κB (NFκB), nuclear factor of activated T cells (NFAT), and inducing mitogen-activated protein kinase pathway. These all highlight BTK as a critical factor in innate and adaptive immunity [24,25]. The prevalence of autoreactive B cells indicates that they depend more on BTK for survival, as evidenced by the loss of autoantibodies when BTK is absent [11]. As BTK is an essential factor in B cell signaling, especially in autoimmune diseases, BTK inhibitors can be a potential treatment for pemphigus (Fig. 3).

A decisive role has been attributed to BTK in the pathogenesis of autoimmune disorders and B-cell malignancies. Different types of B-cell cancer have responded successfully to BTK inhibitors in clinical trials. Also, qualified reactions with BTK inhibitors have been reported in heterogeneous rodent models of arthritis and lupus [14]. In addition, BTK inhibitors were found to be beneficial in treating human systemic lupus erythematosus, rheumatoid arthritis, multiple sclerosis, Sjogren's disease, graft versus host disease, and idiopathic thrombocytopenic purpura [26]. Consequently, BTK inhibitors are under investigation as newly emerged therapeutic options for immunobullous disorders such as pemphigus and pemphigoid disorders.

Two randomized clinical trials were conducted regarding the use of BTK inhibitors in patients with pemphigus. After 24 weeks of both trials, 22% and 18.8% of patients achieved complete remission with rilzabrutinib and tirabruitinib, respectively. However, the complete remission rate was 50% after 52 weeks of tirabrutinib administration. Noteworthy that complete remission was defined as the absence of new blistering or new erythema in both trials. The primary pemphigus area index (PDAI) decreased by 56% with rilzabrutinib and 90% with tirabrutinib after completing the trials (the duration of the trials was not the same). Furthermore, both medications decreased anti-desmoglein levels compared to the baseline; however, it was more evident with tirabrutinib. Meanwhile, tirabrutinib caused no significant effect on total immunoglobulin levels, such as IgG and counts of CD19-positive B-cells within the peripheral blood, noting that tirabrutinib inhibits pathogenic autoantibody titers without affecting total immunoglobulin levels. These results diverge from the observation that patients treated with rituximab for over a year displayed no detectable B cells in their peripheral blood. Additionally, the rate of adverse events with rilzabrutinib was 74%, and 11% of them were reported to be serious (3/27 patients: one cellulitis grade 3, one pneumonia, and one pancreatitis pseudocyst). Nevertheless, pneumonia and pancreatitis pseudocyst were reported to be irrelevant to rilzabrutinib. Moreover, the total incidence of adverse events with tirabrutinib was 87.5%, and none were serious.

Although BTK inhibitors may cause undesirable adverse events such as major hemorrhage, atrial fibrillation, and thrombocytopenia/neutropenia, these side effects often occur with irreversible BTK inhibitors [9,13]. Patients who take reversible BTK inhibitors experience milder adverse effects such as nausea, upper abdominal pain, and headache [13]. Furthermore, tirabruitinib has a low incidence of immunosuppressant-related adverse effects such as hepatic impairment, renal failure, and bone marrow suppression. Notably, reversible BTK inhibitors cause temporary immunosuppression that subsides with tapering and discontinuation. In addition, rilzabrutinib and tirabrutinib allow clinicians to reduce or discontinue CS in the therapeutic regimen of pemphigus patients, which subsequently minimizes and even eliminates CS-related adverse events [4,13]. It is imperative to note that the advantage of BTK treatment lies in the minimal or negligible reduction of B cell counts in the peripheral blood, even with sustained oral administration. In contrast, under rituximab therapy, B cells are often undetectable in the peripheral blood for an extended period exceeding one-year following administration in many cases. Although the therapeutic efficacy of BTK inhibitors in pemphigus may be less pronounced when contrasted with rituximab, it is foreseeable that their continued advancement and exploration will persist in the future. All these findings bold BTK inhibitors as potential therapeutic choices for patients challenging with pemphigus and pemphigoid disorders.

## 5 Conclusion

This review declares that BTK inhibitors are a potent therapeutic option with acceptable safety and efficacy in animal and clinical studies for pemphigus disorder. BTK inhibitors alone or in combination with conventional treatments such as CS were reported to be promising in the management of pemphigus. The adverse reactions associated with reversible BTK inhibitors were not severe and life-threatening.

## Limitation and recommendation

Conducting large-scale randomized controlled clinical trials with long-term follow-ups is necessary to evaluate relapse rate, long-term adverse events, and length of remission in patients with pemphigus. Further studies are needed to compare the safety and efficacy of previous treatments and BTK inhibitors as well as different types of BTK inhibitors.

## Consent for publication

Written informed consent was obtained from the patients for publication of any accompanying images in this study. A copy of the written consent is available for review by the Editor-in-Chief of this journal.

## Funding support

None.

## Transparency declaration

The authors declare that the manuscript is honest, accurate, and transparent. No important aspect of the study is omitted.

## Data availability statement

Data will be made available on request.

## CRediT authorship contribution statement

**Yekta Ghane:** Writing - review & editing, Writing - original draft, Validation, Investigation, Conceptualization. **Nazila Heidari:** Writing - review & editing, Writing - original draft, Validation, Investigation, Conceptualization. **Amirhossein Heidari:** Writing - review & editing, Validation, Investigation, Data curation. **Sara Sadeghi:** Writing - review & editing, Supervision, Project administration. **Azadeh Goodarzi:** Writing - review & editing, Supervision, Project administration.

## Declaration of competing interest

The authors declare that they have no known competing financial interests or personal relationships that could have appeared to influence the work reported in this paper.

## Abbreviations

BCR: B cell receptor
BMX: Bone marrow expressed kinase
BTK: Bruton's tyrosine kinase
CDA: control of disease activity
CLL: Chronic lymphocytic leukemia
cPDAI: Canine Pemphigus Disease Activity Index
CS: Corticosteroid
Dsc: Desmocollin
Dsg: Desmoglein
Ig: Immunoglobulin; Intravenous immunoglobulins
ITK: Interleukin-2-inducible T cell kinase
JAK: Janus kinase
NFAT: Nuclear factor of activated T cell
NFκB: Nuclear factor κB
PF: pemphigus foliaceous
PH: Pleckstrin homology
PIP3: Phosphatidylinositol three phosphate
PLCγ2: Phospholipase Cγ2
PNP: Paraneoplastic pemphigus
PV: Pemphigus Vulgaris
RLK: Resting lymphocyte kinase
TEC: Tyrosine kinase expressed in hepatocellular carcinoma
TNF-α: Tumor factor necrosis alpha
IVIG: Intravenous Immunoglobulin

## References

[1] Didona D. (2019). Pemphigus: current and future therapeutic strategies. Front. Immunol..

[2] Costan V.V. (2021). Comprehensive review on the pathophysiology, clinical variants and management of pemphigus (review). Exp. Ther. Med..

[3] Drucker A.M., Shear N.H. (2021). Bruton tyrosine kinase inhibition warrants further study for pemphigus. Br. J. Dermatol..

[4] Yamagami J. (2021). A multicenter, open-label, uncontrolled, single-arm phase 2 study of tirabrutinib, an oral Bruton's tyrosine kinase inhibitor, in pemphigus. J. Dermatol. Sci..

[5] Tavakolpour S. (2018). Tofacitinib as the potent treatment for refractory pemphigus: a possible alternative treatment for pemphigus. Dermatol. Ther..

[6] Murrell D.F. (2021). Proof of concept for the clinical effects of oral rilzabrutinib, the first Bruton tyrosine kinase inhibitor for pemphigus vulgaris: the phase II BELIEVE study*. Br. J. Dermatol..

[7] Owens T.D. (2022). Discovery of reversible covalent Bruton’s tyrosine kinase inhibitors PRN473 and PRN1008 (rilzabrutinib). J. Med. Chem..

[8] Langrish C.L. (2021). Preclinical efficacy and anti-inflammatory mechanisms of action of the bruton tyrosine kinase inhibitor rilzabrutinib for immune-mediated disease. J. Immunol..

[9] Lee A. (2017). Successful use of Bruton's kinase inhibitor, ibrutinib, to control paraneoplastic pemphigus in a patient with paraneoplastic autoimmune multiorgan syndrome and chronic lymphocytic leukaemia. Australas. J. Dermatol..

[10] Ito Y. (2018). Paraneoplastic pemphigus associated with B-cell chronic lymphocytic leukemia treated with ibrutinib and rituximab. Intern. Med..

[11] Goodale E.C. (2020). Efficacy of a Bruton's Tyrosine Kinase Inhibitor (PRN-473) in the treatment of canine pemphigus foliaceus. Vet. Dermatol..

[12] Goodale E.C. (2020). Open trial of Bruton's tyrosine kinase inhibitor (PRN1008) in the treatment of canine pemphigus foliaceus. Vet. Dermatol..

[13] Murrell D.F. (2021). Proof of concept for the clinical effects of oral rilzabrutinib, the first Bruton tyrosine kinase inhibitor for pemphigus vulgaris: the phase II BELIEVE study. Br. J. Dermatol..

[14] Naik P.P. (2022). Translational autoimmunity in pemphigus and the role of novel Bruton tyrosine kinase inhibitors. J. Transl. Autoimmun..

[15] Pal Singh S., Dammeijer F., Hendriks R.W. (2018). Role of Bruton's tyrosine kinase in B cells and malignancies. Mol. Cancer.

[16] Smith P.F. (2017). A phase I trial of PRN1008, a novel reversible covalent inhibitor of Bruton's tyrosine kinase, in healthy volunteers. Br. J. Clin. Pharmacol..

[17] Yuko A. (2019). Bruton’s tyrosine kinase (Btk) inhibitor tirabrutinib prevents the development of murine lupus. Eur. Exp. Biol..

[18] Malik A.M. (2021). An updated review of pemphigus diseases. Medicina (Kaunas).

[19] Amagai M. (2014). Japanese guidelines for the management of pemphigus. J. Dermatol..

[20] Murrell D.F. (2008). Consensus statement on definitions of disease, end points, and therapeutic response for pemphigus. J. Am. Acad. Dermatol..

[21] Chen D.M. (2020). Rituximab is an effective treatment in patients with pemphigus vulgaris and demonstrates a steroid-sparing effect. Br. J. Dermatol..

[22] Gregoriou S. (2015). Management of pemphigus vulgaris: challenges and solutions. Clin. Cosmet. Invest. Dermatol..

[23] Mano H. (1999). Tec family of protein-tyrosine kinases: an overview of their structure and function. Cytokine Growth Factor Rev..

[24] Aalipour A., Advani R.H. (2014). Bruton's tyrosine kinase inhibitors and their clinical potential in the treatment of B-cell malignancies: focus on ibrutinib. Ther. Adv. Hematol..

[25] Yamagami J. (2022). B-cell targeted therapy of pemphigus. J. Dermatol..

[26] Ringheim G.E., Wampole M., Oberoi K. (2021). Bruton's tyrosine kinase (BTK) inhibitors and autoimmune diseases: making sense of BTK inhibitor specificity profiles and recent clinical trial successes and failures. Front. Immunol..

